# Histopathology and genetic susceptibility in COVID‐19 pneumonia

**DOI:** 10.1111/eci.13259

**Published:** 2020-06-27

**Authors:** Jan von der Thüsen, Menno van der Eerden

**Affiliations:** ^1^ Department of Pathology Erasmus MC Rotterdam The Netherlands; ^2^ Department of Pulmonology Erasmus MC Rotterdam The Netherlands

**Keywords:** COVID‐19, genetics, pulmonary histopathology

## Abstract

**Background:**

The clinical features of COVID‐19 pneumonia range from a mild illness to patients with a very severe illness with acute hypoxemic respiratory failure requiring ventilation and Intensive Care Unit admission.

**Aims:**

To provide a brief overview of the existing evidence for such differences in host response and outcome, and generate hypotheses for divergent patterns and avenues for future research, by highlighting similarities and differences in histopathological appearance between COVID‐19 and influenza as well as previous coronavirus outbreaks, and by discussing predisposition through genetics and underlying disease.

**Materials and Method:**

We assessed the available early literature for histopathological patterns of COVID‐19 pneumonia and underlying risk factors.

**Result:**

The histopathological spectrum of COVID‐19 pneumonia includes variable patterns of epithelial damage, vascular complications, fibrosis and inflammation. Risk factors for a fatal disease include older age, respiratory disease, diabetes mellitus, obesity and hypertension.

**Discussion:**

While some risk factors and their potential role in COVID‐19 pneumonia are increasingly recognized, little is known about the mechanisms behind episodes of sudden deterioration or the infrequent idiosyncratic clinical demise in otherwise healthy and young subjects.

**Conclusion:**

The answer to many of the remaining questions regarding COVID‐19 pneumonia pathogenesis may in time be provided by genotyping as well careful clinical, serological, radiological and histopathological phenotyping.

## 1 INTRODUCTION

Due to the outbreak of the COVID‐19 pandemic, the first quarter of 2020 has seen levels of burden on healthcare systems which have not been seen in relation to a viral infection since the influenza pandemic of 1918.^1^, ^2^, ^3^ While social distancing, testing and containment have undoubtedly led to a significant reduction in morbidity and loss of life, their societal impact and resulting economic upheaval are unprecedented. The extent of the measures taken across the globe varies significantly and ranges from stringent testing and isolation to more lenient ‘intelligent lockdowns’, and these by‐and‐large reflect national and regional cultural and ethical differences in weighing the acceptability of limitation of personal freedoms against strain on the healthcare system and ensuing loss of life, as well as the level of preparedness to accept the consequences of a potential ‘second wave’ in case of a delay in the development of an effective vaccine. However, differences in practice are probably also related to our as yet limited understanding of the natural evolution of the disease, which varies greatly between populations, and is probably related to a host of patient‐related and environmental risk factors.

The clinical features of a patient with COVID‐19 range from a mild illness with slight complaints such as sore throat and headache, to patients who have to be admitted to hospital because of hypoxaemia caused by pneumonia, to patients with a very severe illness who have acute hypoxemic respiratory failure and need to be admitted to an intensive care unit.^3^, ^4^, ^5^, ^6^, ^7^ In general, mortality rates are approximately 2%, but once admitted to hospital this rate can increase to 28%.^4^, ^6^, ^7^ Risk factors for a fatal disease are older age, an increased D‐dimer and an increased SOFA score.^4^ Until now, there are no evidence‐based effective treatment options for COVID‐19. Therefore, hypotheses need to be generated about the underlying pathophysiological mechanisms of a mild to very severe disease, so that new treatment strategies for these different categories of patients can be developed and investigated.

Thus, while we now know that higher age and a range of comorbidities (such as respiratory disorders, cardiovascular disease, diabetes and obesity) pose significant risks for those infected with SARS‐CoV‐2, little is known about the mechanisms behind the infrequent idiosyncratic clinical demise in otherwise healthy young subjects (although there may be an effect of the level of exposure) and the reasons for observed differences in resolution of the disease following an otherwise comparable clinical course in patients with similar risk factor profiles. As in other diseases, the answer to some of these questions may in time be provided by genotyping as well careful clinical, serological, radiological and histopathological phenotyping, which enable mechanistic insights into the differences in pathogenesis and underlying immunological and tissue regenerative response patterns. While little is known so far regarding COVID‐19 histology, we will aim to provide a brief overview of the existing evidence for such differences in host response and outcome, and generate hypotheses for divergent patterns and avenues for future research, by highlighting similarities and differences in histopathological appearance between COVID19 and influenza as well as previous coronavirus outbreaks, and by discussing predisposition through genetics and underlying disease.

## 2 RESPIRATORY TRACT HISTOPATHOLOGY IN INFLUENZA

The archetypical previous pandemic leading to a massive worldwide loss of life due to pneumonia and respiratory failure is the influenza pandemic of 1918, which killed approximately 50 million people worldwide, and which was caused by an RNA virus of the family orthomyxoviridae that contains a negative‐sense (as opposed to the positive strand in coronaviruses), single‐stranded, segmented RNA genome. The histopathology described in cases of various influenza pandemics stems mainly from autopsy cases and therefore leads to bias towards severe cases. Larger case series are available for the 1918 pandemic, as well as limited descriptions of later pandemics and inter‐pandemic cases, and only three autopsy cases of the more recent H5N1 highly pathogenic avian influenza infection.^8^ Nonetheless, it has become clear that while there is a wide range of pulmonary histological response patterns to influenza infection, which varies with both clinical picture and length of the disease course before death, the range appears to be similar for most influenza subtypes. The respiratory tract histology of influenza reflects its cellular tropism, as influenza virus replicates in respiratory epithelial cells throughout the respiratory tree, with nonfatal infections predominantly involving the upper respiratory tract and trachea, but fatal cases of influenza usually result from pneumonia. Coincident or secondary bacterial pneumonias are extremely common in severe influenza and also complicate the histopathological appearance. As these are primarily related to outcome, we will restrict our commentary to the histopathological changes observed in the lower respiratory tract. Changes in the smaller airways include necrosis and complete loss of the epithelial layer (both ciliated and goblet cells), often resulting in the formation of hyaline membranes at these sites. Neutrophilic inflammation may be present in the lumen and extend into surrounding alveoli. In addition, a more chronic lymphoplasmahistiocytic infiltrate is often seen in influenza‐infected airways. The parenchyma shows evidence of acute injury with interstitial congestion, oedema and inflammation (predominantly neutrophilic with some eosinophils), as well as desquamated alveolar epithelial cells, intra‐alveolar oedema, intra‐alveolar haemorrhage, fibrin, hyaline membrane formation and sometimes necrosis of the alveolar septa (necrotizing alveolitis), the latter possibly resulting from frequently observed vascular changes with capillary congestion and thrombosis. Mitotic activity and regeneration of respiratory epithelium start after approximately 5 days and the level of regeneration is possibly related to outcome. Thus, variable mitotic activity but no evidence of true regeneration of the epithelial layer has been seen in rapidly lethal cases. If the patient survives, organizing diffuse alveolar damage, increased numbers of intra‐alveolar macrophages, epithelial regeneration (incl. type II alveolar hyperplasia), squamous metaplasia, chronic interstitial inflammation and eventually interstitial fibrosis may be seen. Megakaryocytes lying within the capillary bed are also commonly seen. Coincident bacterial pneumonias frequently occur and complicate the pathologic picture by massive infiltration of neutrophils into alveolar spaces. In general, most inter‐pandemic cases seem to be associated with fewer changes of primary influenza virus pneumonia (such as oedema), and more changes attributable to secondary bacterial pneumonia.

## 3 RESPIRATORY TRACT HISTOPATHOLOGY IN CORONAVIRUS INFECTION (COVID‐19, SARS, MERS)

**FIGURE 1:**
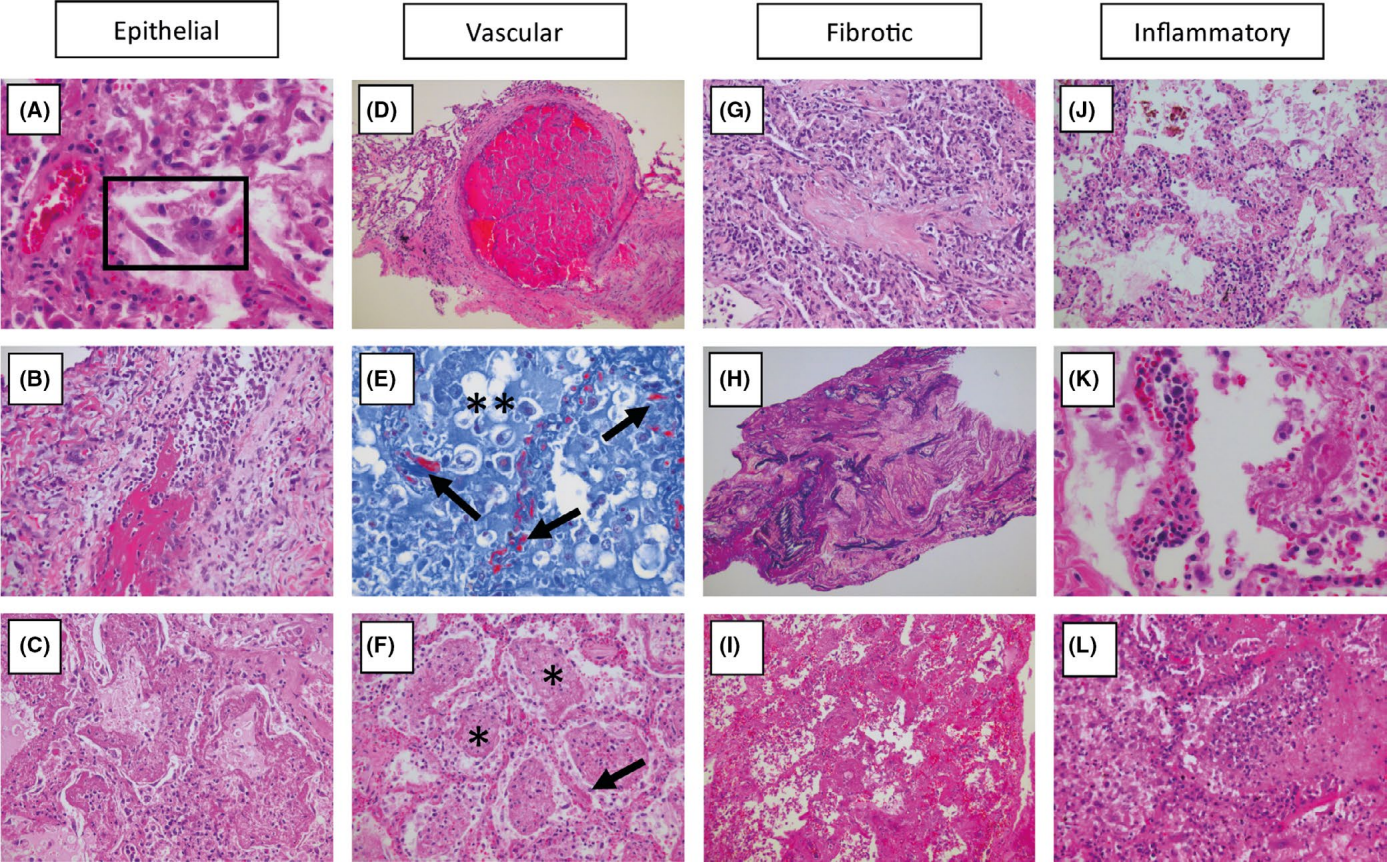
Spectrum of tissue response patterns in COVID‐19 pneumonia. In the lung tissue of patients who died of respiratory failure due to COVID19 pneumonia, there can be evidence of epithelial infection with cytopathic effects of pneumocytes (A, box), denudation of bronchiolar epithelium (B) and evidence of diffuse alveolar damage (DAD) with hyaline membrane formation with organization (C). Observed vascular changes include extensive bilateral and diffuse (micro)vascular damage and its sequelae, with arterial thrombosis with organization (D), microvascular fibrinoid change with hyaline thrombi (E, fibrin‐Lendrum (MSB) stain, arrows) and oedema (**), and extensive intra‐alveolar fibrinous aggregates (F) with an acute fibrinous and organizing pneumonia (AFOP) pattern (F, *). Fibrotic changes vary in appearance and include organizing pneumonia with progression to fibrosis (G), intra‐alveolar fibroelastosis (Elastic‐van Gieson, H) and fibrotic nonspecific interstitial pneumonia (F‐NSIP; I). There is often relatively sparse interstitial chronic inflammation with an acute interstitial pneumonia pattern (AIP; J), foci of lymphocytic vasculitis (K) and acute inflammation, especially in relation to areas of necrosis and possible secondary infection (L)

**FIGURE 2:**
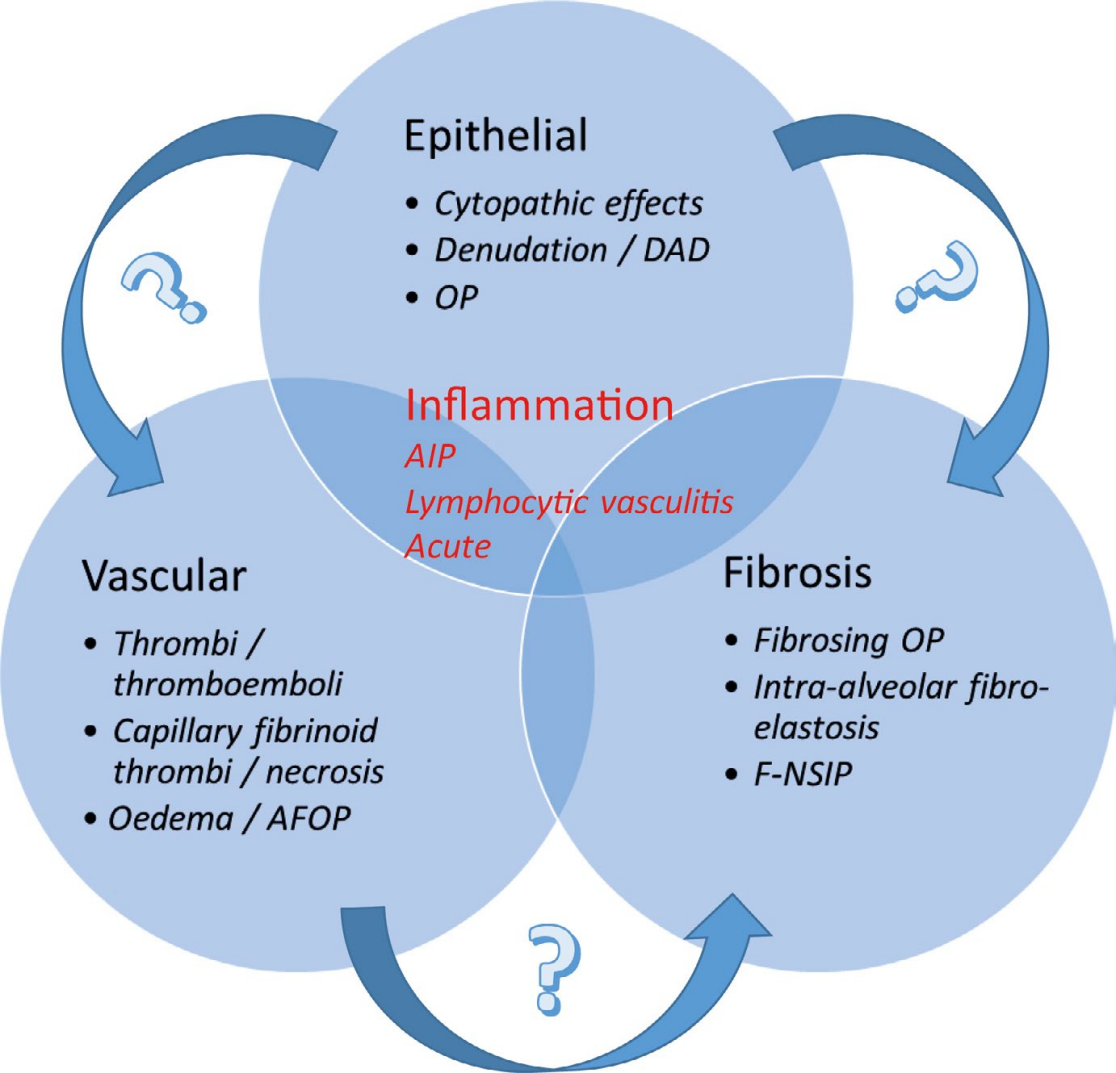
Co‐occurrence and potential progression of tissue response patterns in COVID‐19 pneumonia. AFOP, acute fibrinous and organizing pneumonia; AIP, acute interstitial pneumonia; DAD, diffuse alveolar damage; F‐NSIP, fibrotic nonspecific interstitial pneumonia; OP, organizing pneumonia

Limited data are available regarding the histopathological spectrum of COVID‐19 pneumonia. Of the 41 SARS‐CoV‐2‐infected patients admitted in the early stages of the outbreak, six died from acute respiratory distress syndrome (ARDS),^2^ and four subsequent reports^9^, ^10^, ^11^, ^12^ on the histological examination of the lungs of in total seven patients with SARS‐CoV‐2 indeed showed signs compatible with ARDS. These included bilateral acute changes with diffuse alveolar damage (DAD) with vascular congestion, intra‐alveolar oedema, haemorrhage, proteinaceous exudate, macrophages, denudation and reactive hyperplasia of pneumocytes, patchy inflammatory cellular infiltration and multinucleated giant cells, but hyaline membrane formation was not prominent. The infiltrate consisted of lymphocytes (mostly CD4‐positive), eosinophils and neutrophils. Hyaline thrombi were found in microvessels. We have recently also observed similar changes in COVID‐19 autopsy cases, with evidence of extensive microvascular damage and thrombotic occlusion as the foremost pattern of injury, resulting in intra‐alveolar fibrinous exudates akin to acute fibrinous and organizing pneumonia (AFOP) (Figure 1). This spectrum of abnormalities is highly reminiscent of changes seen in the context of the chronic lung allograft rejection (CLAD) of the restrictive phenotype (restrictive allograft syndrome (RAS)), which we have recently described, and which is presumed to occur secondary to antibody‐mediated endothelial damage and complement activation of humoral rejection episodes.^13^ Such vascular changes are likely to play a central role in the pathogenesis of COVID‐19 and may in part be attributable to dysregulation of the endothelial ACE2 receptor with ensuing bradykinin‐dependent local lung oedema,^14^ as well as a highly pro‐thrombotic state, which can manifest itself in the lung as well as in extra‐pulmonary tissues.^15^ Direct infection of endothelial cells may play a central role in this process.^16^ More chronic changes included intra‐alveolar organization with fibroblastic proliferation and diffuse fibrotic thickening of alveolar walls, consisting of proliferating interstitial fibroblasts. In RAS, we have found this to eventually result in chronic fibrotic patterns of intra‐alveolar fibroelastosis and nonspecific interstitial pneumonia (NSIP).^13^ Time will tell if a similar progression to fibrosis will take place in a subset of survivors of severe COVID‐19, and whether this is indeed secondary to vascular damage, primarily related to the initial epithelial infection, or a combination of these response patterns (Figure 2). Apart from the prominent microvascular changes, the pathological features of COVID‐19 thereby appear to resemble those seen in SARS and Middle Eastern respiratory syndrome (MERS) coronavirus infection.^17^, ^18^, ^19^ Thus, early SARS‐CoV infections were found to be typified by acute diffuse alveolar damage and late stages by a combination of diffuse alveolar damage and acute fibrinous and organizing pneumonia. MERS‐CoV infections also showed exudative diffuse alveolar damage with hyaline membranes, pulmonary oedema, type II pneumocyte hyperplasia, (lymphocytic) interstitial pneumonia and multinucleated syncytial cells.

## 4 BEDSIDE OBSERVATIONS AND FUTURE GUIDANCE IN COVID‐19: TRANSLATION FROM HISTOPATHOLOGY

Poor oxygenation requiring ventilation and oxygen administration in influenza as well as coronavirus infection is likely to be in part directly related to decreased diffusion capacity due to parenchymal destruction as well as increased diffusion distances due to (a) intra‐alveolar aggregates of oedema, fibrin, hyaline membranes, cells and organization, (b) widening of alveolar septa due to oedema and inflammation, (c) vascular changes with capillary congestion and thrombosis and (d) eventually, intrabronchiolar, intra‐alveolar and interstitial fibrosis. While regeneration and reversal of the first three phenomena is theoretically possible, and as in influenza may be related to the regenerative capacity of the host, it may occur at lower rates in SARS‐CoV‐2 infection as compared to other viral pneumonias. Intra‐alveolar and interstitial fibrosis, however, are most likely irreversible and may have contributed to the remarkably high number of patients who cannot be weaned off ventilation in the current COVID‐19 outbreak. Also, in survivors we may eventually see a significant number of cases with irreversible fibrotic lung damage and limited pulmonary function, which has previously been termed COLD (‘Corona Obstructive Lung Disease’). To which extent this term truly reflects the underlying physiology remains to be seen, as the interstitial fibrotic changes which have thus far been observed are likely to also lead to a restrictive lung defect as well as permanently decreased diffusion capacity, and are thus more akin to other types of (idiopathic) interstitial lung fibrosis, such as forms of the abovementioned intra‐alveolar fibroelastosis and fibrotic NSIP, and could therefore perhaps be more accurately termed *Corona‐Associated Lung Disease* (‘CALD’). This will require multi‐centre studies which correlate histopathological findings with clinical and radiological data.

## 5 GENETICS IN COVID‐19

A sudden demise in COVID‐19 may be related to a cytokine storm, which can be an important component of ARDS, multiple organ failure and eventual death in SARS‐CoV‐2, SARS‐CoV and MERS‐CoV infections,^9^ and is reflected by the release of large amounts of pro‐inflammatory cytokines (incl. IFN‐α, IFN‐γ, IL‐1β, IL‐6, IL‐12, IL‐18, IL‐33, TNF‐α, TGFβ) and chemokines (incl. CCL2, CCL3, CCL5, CXCL8, CXCL9, CXCL10, etc) by immune effector cells.^12^ In the case of influenza, hypercytokinaemia has been found to be correlated with the occurrence of haemophagocytosis, which was observed to a varying degree in cases of influenza pneumonia. Whether a similar mechanism is at play in COVID‐19 has yet to be resolved from haematological, immunological and histopathological assessment. In general, however, an overly active inflammatory response is likely to contribute to tissue damage and systemic effects in COVID‐19, and this may be related to genetically determined differences in inherent individual immune response mechanisms as well as being subject to the influence of concomitant diseases. Thus, previous research shows that numerous HLA polymorphisms correlate with the susceptibility of SARS‐CoV and MERS‐CoV infection, and gene polymorphisms of MBL (mannose‐binding lectin) associated with antigen presentation have also been linked to the risk of SARS‐CoV infection.^20^ Also, the single‐nucleotide polymorphism rs12252‐C/C in the gene IFITM3 (which encodes interferon‐induced transmembrane protein 3) has been demonstrated to be a risk factor for severe influenza, and has also been found in a patient with COVID‐19.^21^ Genetics may also play a role through polymorphisms of genes which encode for (a) proteins that are exploited by SARS‐CoV‐2, such as the highly conserved angiotensin‐converting enzyme 2 (ACE2), which, as was also the case for SARS‐CoV, it uses for docking and cellular entry in respiratory cells, or (b) proteins which provide protection against the effects of the virus (such as surfactant proteins, but also ACE2 itself).^22^ Genetic association studies are currently ongoing to unravel such hypotheses and have found correlations between ACE2 variants and COVID‐19 susceptibility.^23^, ^24^ Interestingly, as an X‐linked phenotype, the effectiveness of interaction‐booster and interaction‐inhibitor variants of ACE2 can be more definite in males than females, and could contribute towards a higher mortality rate in males, accounting for up to ∼70% of death caused by SARS‐CoV2, SARS‐CoV or MERS‐CoV,^25^ in addition to a likely higher rate of risk factors (eg smoking) and a possibly different immune response in males. Other entry mechanisms may also play a role, such as transmembrane protease serine 2 (TMPRSS2), and TMPRSS2 variants and expression have indeed been linked to differences in COVID‐19 severity.^26^ Susceptibility to the development of post–COVID‐19 pulmonary fibrosis could also have a genetic component, as variants in numerous genes or their promotors (such as MUC5B and TERT) have been found to predispose to lung fibrosis, both in the context of idiopathic pulmonary fibrosis and in interstitial fibrosis related to hypersensitivity pneumonitis and collagen vascular disease.^27^, ^28^, ^29^, ^30^

## 6 UNDERLYING DISEASE IN COVID‐19

ACE2 could also be pivotal in the particular susceptibility of diabetic and hypertensive patients for fulminant COVID‐19. While diabetic patients are known to have impaired immune responses, diabetes (as well as hypertension) are associated with activation of the renin‐angiotensin system in different tissues and are often treated with ACE inhibitors and angiotensin receptor blockers (ARBs), which can lead to increased expression of ACE2, thereby potentially facilitating viral uptake. Similarly, in MERS, dipeptidyl peptidase 4 (DPP4) inhibition in diabetic patients could have potentiated entry of the virus MERS‐CoV, which uses DPP4 as its receptor.^31^ Discontinuation of ARBs would be premature at this stage, however, as ACE2 is also likely to protect against the effects of SARS‐CoV‐2 infection.^32^, ^33^, ^34^ Obesity is a further important risk factor for mortality following COVID‐19 and may be related to the fact that the majority of those with obesity also have impaired lung function and underlying diseases, such as chronic lung disease, including asthma, cardiac problems or diabetes, which are risk factors on their own for survival following ICU admission, but also for mortality in COVID‐19, in which multi‐organ failure is a common occurrence and good baseline cardiorespiratory function of crucial benefit.^35^

## 7 CONCLUSION

In summary, despite the relatively recent outbreak of the COVID‐19 pandemic, an impressive body of preliminary evidence has already been accumulated which points to a remarkable heterogeneity of disease patterns from a clinical, radiological and histopathological point of view. This has identified broad risk groups, but sometimes also idiosyncratic responses of individual patients in these groups are seen. These may be in part related to underlying genetic variations, which contribute to differences in outcome in the short term, but possibly also affect the extent of residual pulmonary damage following survival of the acute phase of the disease. More research is required to expand and confirm these associations, in order to enable adequate prevention, prognosis, follow‐up and possibly treatment in at‐risk populations.

## CONFLICT OF INTEREST

The authors have not received funding for this publication and have nothing to disclose.

## References

[1] Wu F , Zhao SU , Yu B , et al. A new coronavirus associated with human respiratory disease in China. Nature. 2020;579(7798):265‐269. 32015508 10.1038/s41586-020-2008-3 PMC7094943

[2] Huang C , Wang Y , Li X , et al. Clinical features of patients infected with 2019 novel coronavirus in Wuhan. China. Lancet. 2020;395:497‐506. 31986264 10.1016/S0140-6736(20)30183-5 PMC7159299

[3] Chan J‐W , Yuan S , Kok K‐H , et al. A familial cluster of pneumonia associated with the 2019 novel coronavirus indicating person‐to‐person transmission: a study of a family cluster. Lancet. 2020;395:514‐523. 31986261 10.1016/S0140-6736(20)30154-9 PMC7159286

[4] Zhou F , Yu T , Du R , et al. Clinical course and risk factors for mortality of adult inpatients with COVID‐19 in Wuhan, China: a retrospective cohort study. Lancet. 2020;395:1054‐1062. 32171076 10.1016/S0140-6736(20)30566-3 PMC7270627

[5] Chen N , Zhou M , Dong X , et al. Epidemiological and clinical characteristics of 99 cases of 2019 novel coronavirus pneumonia in Wuhan, China: a descriptive study. Lancet. 2020;395:507‐513. 32007143 10.1016/S0140-6736(20)30211-7 PMC7135076

[6] Xu X‐W , Wu X‐X , Jiang X‐G , et al. Clinical findings in a group of patients infected with the 2019 novel coronavirus (SARS‐Cov‐2) outside of Wuhan, China: retrospective case series. BMJ. 2020;368:m606. 32075786 10.1136/bmj.m606 PMC7224340

[7] Guan W‐J , Ni Z‐Y , Hu YU , et al. Clinical characteristics of coronavirus disease 2019 in China. N Engl J Med. 2020;382(18):1708‐1720. 32109013 10.1056/NEJMoa2002032 PMC7092819

[8] Taubenberger JK , Morens DM . The pathology of influenza virus infections. Annu Rev Pathol. 2008;3:499‐522. 18039138 10.1146/annurev.pathmechdis.3.121806.154316 PMC2504709

[9] Xu Z , Shi L , Wang Y , et al. Pathological findings of COVID‐19 associated with acute respiratory distress syndrome. Lancet Respir Med. 2020;8:420‐422. 32085846 10.1016/S2213-2600(20)30076-X PMC7164771

[10] Tian S , Hu W , Niu LI , et al. Pulmonary pathology of early‐phase 2019 novel coronavirus (COVID‐19) pneumonia in two patients with lung cancer. J Thorac Oncol. . 2020;15(5):700‐704. 32114094 10.1016/j.jtho.2020.02.010 PMC7128866

[11] Zhang H , Zhou P , Wei Y , et al. Histopathologic changes and SARS‐CoV‐2 immunostaining in the lung of a patient with COVID‐19. Ann Intern Med. 2020;172(9):629. 32163542 10.7326/M20-0533 PMC7081173

[12] Yao XH , Li TY , He ZC , et al. A pathological report of three COVID‐19 cases by minimally invasive autopsies. Zhonghua Bing Li Xue Za Zhi. 2020;49:E009. 10.3760/cma.j.cn112151-20200312-00193 32172546

[13] von der Thüsen JH , Vandermeulen E , Vos R , et al. The histomorphological spectrum of restrictive chronic lung allograft dysfunction and implications for prognosis. Mod Pathol. 2018;31:780‐790. 29327719 10.1038/modpathol.2017.180

[14] Klok FA , Kruip MJHA , van der Meer NJM , et al. Incidence of thrombotic complications in critically ill ICU patients with COVID‐19. Thromb Res. 2020;191:145‐147. 32291094 10.1016/j.thromres.2020.04.013 PMC7146714

[15] van de Veerdonk F , Netea MG , van Deuren M , et al. Kallikrein-kinin blockade in patients with COVID‐19 to prevent acute respiratory distress syndrome. Elife. 2020; 9: e57555. 32338605 10.7554/eLife.57555 PMC7213974

[16] Varga Z , Flammer AJ , Steiger P , et al. Endothelial cell infection and endotheliitis in COVID‐19. Lancet. 2020;395(10234):1417‐1418. 32325026 10.1016/S0140-6736(20)30937-5 PMC7172722

[17] Ding Y , Wang H , Shen H , et al. The clinical pathology of severe acute respiratory syndrome (SARS): a report from China. J Pathol. 2003;200:282‐289. 12845623 10.1002/path.1440 PMC7168017

[18] Ng DL , Al Hosani F , Keating MK , et al. Clinicopathologic, immunohistochemical, and ultrastructural findings of a fatal case of Middle East respiratory syndrome coronavirus infection in the United Arab Emirates, April 2014. Am J Pathol. 2016;186:652‐658. 26857507 10.1016/j.ajpath.2015.10.024 PMC7093852

[19] Liu J , Zheng X , Tong Q , et al. Overlapping and discrete aspects of the pathology and pathogenesis of the emerging human pathogenic coronaviruses SARS‐CoV, MERS‐CoV, and 2019‐nCoV. J Med Virol. 2020;92:491‐494. 32056249 10.1002/jmv.25709 PMC7166760

[20] Li X , Geng M , Peng Y , et al. Molecular immune pathogenesis and diagnosis of COVID‐19. Journal of Pharmaceutical Analysis. 2020;10(2):102‐108. 32282863 10.1016/j.jpha.2020.03.001 PMC7104082

[21] Thevarajan I , Nguyen THO , Koutsakos M , et al. Breadth of concomitant immune responses prior to patient recovery: a case report of non‐severe COVID‐19. Nat Med. 2020;26(4):453‐455. 32284614 10.1038/s41591-020-0819-2 PMC7095036

[22] Guzzi PH , Mercatelli D , Ceraolo C , Giorgi FM . Master regulator analysis of the SARS‐CoV‐2/human interactome. J Clin Med. 2020;9(4):982. 10.3390/jcm9040982 PMC7230814 32244779

[23] Renieri A , Benetti E , Tita R , et al. ACE2 variants underlie interindividual variability and susceptibility to COVID‐19 in Italian population. MedRxiv. 2020. 10.1101/2020.04.03.20047977 PMC7366459 32681121

[24] Delanghe JR , Speeckaert MM , De Buyzere ML . The host's angiotensin‐converting enzyme polymorphism may explain epidemiological findings in COVID‐19 infections. Clin Chim Acta. 2020;505:192‐193. 32220422 10.1016/j.cca.2020.03.031 PMC7102561

[25] Darbani B . The expression and polymorphism of entry machinery for COVID‐19 in human: juxtaposing population groups, gender, and different tissues. Int J Res Public Health. 2020;17(10):3433. 10.3390/ijerph17103433 PMC7277542 32423095

[26] Asselta R , Paraboschi EM , Mantovani A , et al. TMPRSS2 variants and expression as candidates to sex and country differences in COVID‐19 severity in Italy. Aging (Albany). 2020;12:10087‐10097. 10.18632/aging.103415 PMC7346072 32501810

[27] Borie R , Le Guen P , Ghanem M , et al. The genetics of interstitial lung diseases. Eur Respir Rev. 2019;28(153):190053. 31554702 10.1183/16000617.0053-2019 PMC9488931

[28] Ley B , Torgerson DG , Oldham JM , et al. Rare protein‐altering telomere‐related gene variants in patients with chronic hypersensitivity pneumonitis. Am J Respir Crit Care Med. 2019;200:1154‐1163. 31268371 10.1164/rccm.201902-0360OC PMC6888660

[29] Ley B , Newton CA , Arnould I , et al. The MUC5B promoter polymorphism and telomere length in patients with chronic hypersensitivity pneumonitis: an observational cohort‐control study. Lancet Respir Med. 2017;5:639‐647. 28648751 10.1016/S2213-2600(17)30216-3 PMC5555581

[30] Stock CJW , De Lauretis A , Visca D , et al. Defining genetic risk factors for scleroderma‐associated interstitial lung disease : IRF5 and STAT4 gene variants are associated with scleroderma while STAT4 is protective against scleroderma‐associated interstitial lung disease. Clin Rheumatol. 2020;39:1173‐1179. 31916109 10.1007/s10067-019-04922-6 PMC7142048

[31] Iacobellis G . COVID‐19 and diabetes: Can DPP4 inhibition play a role? Diabet Res Clin Pract. 2020;162:108125. 10.1016/j.diabres.2020.108125 PMC7271223 32224164

[32] Ma RCW , Holt RIG . COVID‐19 and diabetes. Diabet Med. 2020;37(5):723‐725. 32242990 10.1111/dme.14300 PMC7228343

[33] Pal R , Bhansali A . COVID‐19, diabetes mellitus and ACE2: The conundrum. Diabet Res Clin Pract. 2020;162:108132. 10.1016/j.diabres.2020.108132 PMC7118535 32234504

[34] de Simone G . ESC Council on Hypertension. Position Statement of the ESC Council on hypertension on ACE‐inhibitors and angiotensin receptor blockers. Eur Soc Cardiol. 2020. Available at https://www.escardio.org/Councils/Council-on-Hypertension-(CHT)/News/position-statement-of-the-esc-council-on-hypertension-on-ace-inhibitors-and-ang).

[35] Dietz W , Santos‐Burgoa C . Obesity and its implications for COVID‐19 mortality. Obesity (Silver Spring). 2020;28(6):1005. 32237206 10.1002/oby.22818

